# Editorial: Advances in novel pharmacotherapeutics and drug discovery: computational, experimental, translational, and clinical models

**DOI:** 10.3389/fphar.2024.1465835

**Published:** 2024-08-09

**Authors:** Cristian Sandoval, Francisco Torrens, Vanessa Souza-Mello, Jorge Farías

**Affiliations:** ^1^ Escuela de Tecnología Médica, Facultad de Salud, Universidad Santo Tomás, Osorno, Chile; ^2^ Departamento de Medicina Interna, Facultad de Medicina, Universidad de La Frontera, Temuco, Chile; ^3^ Department of Chemical Engineering, Faculty of Engineering and Science, Universidad de La Frontera, Temuco, Chile; ^4^ Institut Universitari de Ciència Molecular, Universitat de València, València, Spain; ^5^ Laboratorio de Morfometría, Metabolismo y Enfermedades Cardiovasculares, Centro Biomédico, Instituto de Biología, Universidade do Estado do Rio de Janeiro, Rio de Janeiro, Brazil

**Keywords:** drug delivery & targeting, public health, cancer, QSAR & docking, multidisciplinary approach

This Research Topic includes contributions to pharmacotherapeutics, as well as the development of new drug discovery and delivery systems. The aim is to enhance the efficiency of screening, identification, development, and advancements in these areas using *in silico*, *in vitro*, and *in vivo* models. This Research Topic consists of a total of 11 papers. Among them, there are 7 original research pieces, 2 literature reviews, and 1 systematic review.

The first article on this Research Topic (Baek et al.) examines the impact of oxidative stress-induced mitochondrial damage on hair cells. It also explores the potential for developing medications that specifically target components of the mitochondrial redox network to address hearing loss pathology. They suggest mitochondria as a key target to protect auditory cells and maintain hearing function, where antioxidants can provide protection against drug-induced ototoxicity. However, because antioxidants are not selective for specific target molecules and can affect multiple signaling pathways simultaneously with low specificity, it is difficult to determine drug efficacy and safety at low concentrations.

As we continue to discuss pharmacological strategies and their potential against various disorders, a number of therapeutic tools and approaches have been tested in clinical trials, in example for the disorder known as Fragile X Syndrome (Milla et al.), which is brought on by the hypermethylation of a CGG repeat expansion in the 5′-untranslated region of the Fragile X Messenger Ribonucleoprotein 1 (FMR1) gene, leading to transcriptional silencing of the gene, which is the most common cause of hereditary intellectual impairments, such as autism spectrum disease. While there is not a cure, treatment and medication can help control symptoms. So far, pharmacological methods have mostly been used to design drugs that change intracellular pathways, change GABA levels, or block mGluR interactions.

Within this Research Topic, we also located original articles evaluating medications for immunoglobulin A nephropathy (Xu et al.), with tetrandrine showing the strongest inhibitory effect against mesangial cell proliferation induced by deglycosylated human IgA1, particularly when contrasted with non-stimulation of deS/deGal IgA. Similar to this, there is a chance of severe kidney damage when using colistin, an antibacterial medication used as a last option. Consequently, it has been discovered that colistin-encapsulated liposomes lessen nephrotoxicity (Mektrirat et al.). In fact, it has been discovered that the application of liposomes shields human embryonic kidney cells from cytotoxicity that is dependent on both time and concentration. It is also important to note that colistin liposomal formulations reduce clinical and pathological nephrotoxicity in rat models, which shows that they could make things safer.

Lung cancer exhibits the highest morbidity rate when compared to other types of cancer and is the primary cause of mortality from malignant neoplasms worldwide. Non-small-cell lung cancer accounts for around 85%–90% of all cases (Reck and Rabe 2017). Cisplatin or other platinum-based combinations have been the most effective systemic chemotherapy for non-small-cell lung cancer for more than 20 years. However, the introduction of targeted therapy and immunotherapy has resulted in changes to the treatment strategies (Saar et al., 2023). Immunotherapies are sophisticated medications that utilize the body’s natural defenses to fight against malignant growths by stimulating or inhibiting various pathways of the immune system. Typical techniques involve the use of PD-1 pathway blocking pharmaceuticals, drugs that hinder the CTLA-4 pathway, and calix pyrroles (Sève and Dumontet, 2005). According to Geretto et al. (2018), Fraisinib has unquestionably demonstrated its efficacy against a variety of tumor cell types, with a focus on its noteworthy performance against non-small-cell lung cancer. Without a doubt, Fraisinib exhibits exceptional anti-tumoral properties with low toxicity in mice (Toumia et al.). Additionally, scientists have discovered and confirmed glycyl-tRNA synthetase as the specific protein that this chemical targets. By blocking the enzyme GARS1, Fraisinib changes a number of important biological processes that are linked to tumor growth, aggressiveness, and invasion.

This idea should also be applied to traditional Chinese medicine. In this field, it is common to use a certain mix of six herbs (pinellia ternata, bran fried Fructus aurantii, ginger, raw bamboo, fried licorice, and raw orange peel) to help people with hyperlipidemia feel better. Oxidative stress indeed has a substantial impact on the progression of hyperlipidemia. A pharmacokinetic and pharmacodynamic model was used in an *in vivo* investigation to investigate the antioxidant activity of Wendan Decoction. The study observed a reduction in plasma triglyceride, total cholesterol, and low-density lipoprotein cholesterol levels, while high-density lipoprotein cholesterol levels varied depending on the dosage (Xu et al.).

We hope that the reader will find this Research Topic a useful reference for the state of the art in the emerging field of pharmacotherapeutics and drug discovery through computational, experimental, translational, and clinical models.
